# Neo-adjuvant chemotherapy plus immunotherapy in resectable N1/N2 NSCLC

**DOI:** 10.1186/s12885-023-11745-x

**Published:** 2023-12-21

**Authors:** Chengli Du, Yunhao Chen, Yuwei Zhou, Difang Zheng, Jiangang Zhao, Jie Tang, Yihe Wu, Zhengliang Tu

**Affiliations:** Department of Thoracic Surgery, the first affiliated hospital, Zhejiang University School, 1367 West Wenyi Rd., Hangzhou, 311121 China

**Keywords:** Locally advanced NSCLC, Immunotherapy, Nodal status, Pathological response, Survivial

## Abstract

**Background:**

Locally advanced non-small cell lung cancer (NSCLC) with N1/N2 lymph node metastasis is challenging with poor survival. Neo-adjuvant chemo-immunotherapy has gained benefits in a proportion of these patients. However no specific biomarker has been proved to predict the effect before therapy. In addition, the relationship of nodal status and survival after neo-adjuvant chemo-immunotherapy is still not well stated.

**Methods:**

A total of 75 resectable NSCLC patients with N1/N2 stage who received neo-adjuvant chemo-immunotherapy plus surgery were retrospectively studied. The clinical characteristics, surgical information and safety parameters were collected. The correlations of major pathological response (MPR) and pathological complete response (pCR) with clinical data were analyzed. The progression free disease(PFS) and overall survival(OS) were evaluated with pathological response and nodal status.

**Results:**

Of the 75 patients, 69 (92%) patients experienced treatment related adverse effects, while grade 3–4 adverse effects occurred in 8 (10%) patients. All the patients received surgical R0 resection with a MPR rate of 60% and a pCR rate of 36%. 67% of N1 patients and 77% of N2 patients had nodal clearance after neo-adjuvant treatment. A significant difference was observed between pathological response with age, histology and multiple lymph node metastasis. The PFS was better in the MPR cohort. The PFS was 90.1% and 83.6% at the nodal clearance group at the time of 12 and 18 months, compared with 70.1% and 63.7% at the nodal residual group.

**Conclusions:**

The neo-adjuvant chemo-immunotherapy for locally advanced NSCLC with nodal positive was safe and feasible. The patients with elder age and squamous-cell carcinoma (SCC) were more likely to have better pathological response, while multiple nodal metastasis was a negative predictor. The clearance of lymph node resulted in significantly longer PFS and OS.

**Supplementary Information:**

The online version contains supplementary material available at 10.1186/s12885-023-11745-x.

## Introduction

Lung cancer is the leading cause of cancer-related deaths in the worldwide, among which 85% are non-small-cell lung cancer (NSCLC) [1]. Approximately 20% of NSCLC are diagnosed in locally advanced stage with N1/N2 lymph node metastasis. The outcome for this subtype of patients remains poor, with 5-year overall survival of 20–40% [2, 3]. Even the chemotherapy is combined with surgery, only 5–6% of 5-year survival is improved [4]. Effective systemic treatments continue to be needed for potentially resectable advanced NSCLC.

The immune checkpoint inhibitors (ICIs), targeting PD-1/PD-L1 axis, can activate the immune system to recognize and kill cancer cells via a T cell-mediated immune response [5]. Even the recent progress has been made with adjuvant immunotherapy for resectable NSCLC [6], the neo-adjuvant mode of immunotherapy provides an early opportunity to treat micrometastatic disease, which has reached a consensus in treating the locally advanced NSCLC [7, 8]. Pathological complete response (pCR) and major pathological response (MPR), strongly associated with better survival, are proposed to be useful surrogates of neo-adjuvant therapy response [9]. Compared with the low proportion of pCR (median 4%) and MPR (median 18%) in the induction chemotherapy in NSCLC, the recent clinical trials show a great improvement by combining immunotherapy with chemotherapy. As reported in CheckMate 816 trial, nivolumab plus chemotherapy is superior to chemotherapy alone in the endpoint of pCR and MPR, as well as EFS and OS [10]. Thus the therapy mode of chemo-immunotherapy has been widely prosed in the neo-adjuvant context. Despite benefits gained in part of patients, its potential safety problems and surgical difficulties also raise concerns. Till now no specific biomarker can accurately predict the survival advantage or pathological response of neo-adjuvant chemo-immunotherapy, including PD-L1 expression or tumor mutation burden. For the locally advanced NSCLC, several studies prove that the clearance of nodal diseases is strongly correlated with the survival benefits after neo-adjuvant chemo-radiotherapy [11]. However no evidence has shown the benefit of nodal clearance in the chemo-immunotherapy therapy mode.

In this retrospective study, we analyzed the clinical data from 75 resectable N1/N2 positive NSCLC after neo-adjuvant chemo-immunotherapy. The objective of the study was to identify predictors of pathological response, as well as exploring the clinical safety, feasibility, and effectiveness. In addition, we also sought to determine the relationship between nodal clearance with survival benefits.

## Methods

### Patients

We retrospectively collected data from 75 resectable NSCLC patients with N1/N2 stage (IIb-IIIb stage) at the First Affiliated Hospital, College of Medicine, Zhejiang University. The patients included had an Eastern Cooperative Oncology Group performance status of 0 or 1, adequate organ function, adequate pulmonary function. Patients with known EGFR mutations or ALK translocations were excluded. All the patients were treated with neo-adjuvant chemotherapy plus immunotherapy and followed by surgery. The timing of surgery was decided by the treating surgeons after 2–4 cycles of neo-adjuvant therapy. This study was approved by the Ethics Committee of First Affiliated Hospital, College of Medicine, Zhejiang University (approval number 2023 − 0598). All patients were informed of the study and consented to the enrollment. All the procedures were conducted in accordance with the Declaration of Helsinki.

### Peri-operative evaluation

The preoperative staging was performed according to the American Joint Committee on Cancer (AJCC) 8th edition [12]. The primary tumors were confirmed by tumor biopsy, and the nodal status was staged by Endobronchial Ultrasound-Guided Transbronchial Needle Aspiration (EBUS-TBNA), positron emission tomography/CT scan (PET-CT) or contrast-enhanced CT. Clinical N positive was diagnosed if their diameters were > 1.5 cm on contrast-enhanced CT.

Radiologic response of tumors was evaluated according to the Response Evaluation Criteria in Solid Tumors (RECIST) version 1.1 [13]. Pathological response was assessed by measuring the percentage of residual viable tumor in primary tumors and nodes as reported [14]. Pathological complete response (pCR) and major pathological response (MPR) were defined as 0% and ≤ 10% residual viable tumor cells in the primary tumors and resected lymph nodes. The treatment-related adverse events were graded according to the National Cancer Institute Common Terminology Criteria for Adverse Events, version 5.0. Surgical complications were documented according to the criteria defined by the Society of Thoracic Surgeons database [15].

### Chemotherapy and immunotherapy

All the patients included were arranged to receive neo-adjuvant chemotherapy and immunotherapy by multidisciplinary discussions. The chemotherapy and ICIs were administered every three weeks. After two cycles of neo-adjuvant therapy, the patients were evaluated to proceed to surgery. If no radiological regression was observed, 1–2 more cycles of treatment were added before surgery. 4 cycles were considered the maximam doses for neo-adjuvant treatment. During the first follow-up visit, adjuvant therapy was scheduled if necessary. In the whole treatment duration, up to 4 cycles of chemotherapy and 1 year of immunotherapy were performed.

### Evaluation of PD-L1

The expression of PD-L1 was analyzed by immunohistochemistry (IHC) using monoclonal mouse anti-human PD-L1 clone 22C3 (Dako, Agilent Technologies, CA, USA) on the pretreatment biopsy samples. The tumor proportion score (TPS) were assessed as the percentage of at least 100 viable tumor cells.

### Statistical analysis

The associations between clinical parameters and pathologic responses were evaluated by using Chi-squared test or Fisher’s test. PFS and OS were estimated using Kaplan–Meier curves and the log-rank test. Statistical analyses were performed using SPSS 25.0 (SPSS, Inc., Chicago, IL, USA). All results tested were two-tailed and were considered statistically significant if the *p* value was less than 0.05.

## Results

### Patients characteristics

From January 2021 to June 2022, 82 patients with resectable N1/N2 NSCLC received neo-adjuvant chemotherapy plus immunotherapy in our center, among which 75 (91.4%) patients underwent definitive surgery. The reasons for surgery cancellation included poor lung function (2 cases), treatment-related adverse events (3 cases), operation refusal by patients (1 case) and disease progression (1 case). The 75 patients, recived neo-adjuvant therapy and surgery, were further retrospectively evaluated in our study. The baseline characteristics of the patients were shown in Table 1. The median age of the patients was 64 years (41–78), 69 patients (92%) were men, and 67 patients (89%) were current or former smokers. For the tumor and lymph node characteristics, the median tumor lesion size was 39 mm (15–67), 26 patients (35%) were N1 positive and 49 patients (65%) were N2 positive. The TNM stage was listed as follows: stage IIb 24 (32%), stage IIIa 41 (55%), stage IIIb 10 (13%). Of the histological subtypes, 60 patients (80%) had squamous-cell carcinoma and 15 patients (20%) had adenocarcinoma.

**Table 1:** Characteristics of the Patients at Baseline

Age	64 (41–67)
Sex	
Male Female	69 (92%) 6 (8%)
Smoking history	
Current/former Never	67 (89%) 8 (11%)
Tumour lesion size, mm	
Median (range)	39 (15–67)
Nodal stage	
N1 N2	26 (35%) 49 (65%)
TNM stage	
IIb IIIa IIIb	24 (32%) 41 (55%) 10 (13%)
Histology	
Squamous-cell carcinoma Adenocarcinoma	60 (80%) 15 (20%)
PD-L1 expression	
< 1% 1–50% ≥ 50% NA	21 (28%) 21 (28%) 10 (13%) 23 (31%)

### Peri-operative treatment and safety

For the immunotherapy in these 75 patients, 52 received tislelizumab and 23 received pembrolizumab. For the chemotherapy regimens, the 60 squamous-cell carcinoma patients were treated with carboplatin plus paclitaxel, while the adenocarcinoma patients used carboplatin with pemetrexed. Most patient received 2 doses of neo-adjuvant therapy (58, 77%), followed by 3 doses (12, 16%) and 4 doses (5, 7%). There were no treatment-related surgical delays except for 1 patient with increased aminotransferases, and the median interval between the last administration and surgery was 32.6 days (24–71). According to RECIST criteria response, 69 (92%) of 75 patients had an overall response, among which 3 (4%) had a complete response and 66 (88%) had a partial response, while 6 (8%) patients had a stable disease. And no progression disease case was observed. All the 75 had curatively radical surgery and 50 patients received at least one cycle of adjuvant chemotherapy, immunotherapy or both. The peri-operative treatment parameters were shown in Table S1 and Table S2.

The treatment-related adverse events were summarized in Table 2. During the peri-operative treatment, 69 (92%) patients experienced at least grade 1–2, while 8 (10%) experienced grade 3–4 adverse events. The most common ones of any grade were anaemia (68%), neutropenia (57%), fatigue (52%) and anorexia (51%). The grade 3–4 adverse events were listed as follows: anaemia (4%), neutropenia (4%) and increased aminotransferases (3%). There was no treatment-related death or surgery cancelation.

**Table 2:** Any treatment-related adverse event

	Grade 1–2	Grade 3–4
**Symptom side effects**		
Fatigue	39(52%)	0
Anorexia	38(51%)	0
Alopecia	28(37%)	0
Rash	23(31%)	0
Nausea	22(29%)	0
Vomiting	19(25%)	0
Diarrhoea	15(20%)	0
Arthralgia	13(17%)	0
Paraesthesia	10(13%)	0
Constipation	6(8%)	0
Pruritus	5(7%)	0
**Abnormal lab examinations**		
Anaemia	51(68%)	3(4%)
Neutropenia	43(57%)	3(4%)
hypothyroidism	20(27%)	0
Increased aminotransferases	18(24%)	2(3%)
Thrombocytopenia	16(21)	0
hyponatremia	10(13%)	0
Increased creatinine	9(12%)	0
hyperuricemia	6(8%)	0
Hyperglycemia	5(7%)	0
Hypocalcemia	3(4%)	0

### Surgical summary and pathological assessment

All the patients enrolled in this study got R0 resection. The surgical information and post-operative complications were shown in Table 3. Of the 75 patients, 51 (68%) had video-assisted thoracoscopic surgery and 24 (32%) had a thoracotomy. The surgical extension preformed were listed as follows: lobectomy (58, 77%), bilobectomy (7, 9%), sleeve lobectomy (8, 11%) and pneumonectomy (2, 3%). Post-operative complications were observed in 22 (29%) patients. The most frequent complications were prolonged air leak (12, 16%), increased pleural effusion duration (8, 11%) and atrial fibrillation (8, 11%).

**Table 3:** Surgical information and pathological status after surgery

Location, cases	
LU LL RU RM RL	22 9 25 3 16
Surgical approach	
VATS Open	51(68%) 24(32%)
Surgical resection	
Lobectomy Bilobectomy Sleeve lobectomy Pneumonectomy	58(77%) 7 (9%) 8 (11%) 2(3%)
Operative time, min	
Median (range)	130(70–240)
Estimated blood loss, ml	
Median (range)	80(20–500)
Length of postoperative hospitalization, days	
Median (range)	5.2(3–16)
Pathological response	
PCR non-PCR mPR non-mPR	27(36%) 48(64%) 45(60%) 30(40%)
Nodal status after neo-adjuvant therapy	
N2 clearance N2 residual N1 clearance N1 residual	34(67%) 15(33%) 20(77%) 6 (23%)
Postoperative complications	
Prolonged air leak > 5 days Pneumonia Pleural effusion duration > 5 days Atrial fibrillation Recurrent nerve paralysis Chylothorax Postoperative bleeding	12(16%) 5 (7%) 8 (11%) 8 (11%) 2 (3%) 1 (1%) 1 (1%)

LU, left upper; LL, left lower; RU, right upper; RM, right middle; RL, right lower; pCR, pathological complete response; MPR, major pathological response

Final pathological evaluation showed that 45 (60%) patients had a major pathological response (MPR), including 27 (36%) patients pathological complete response (pCR) (Table 3). Residual non-viable tumor beds largely consisted of fibrotic, elastostotic, and necrotic tissue mixed with regions of inflammation, foamy histiocytes, and multi-nucleated giant cells (Sup Fig. 1). We also evaluated the pathological status of the lymph node at the time of surgery. Of the 49 N2 positive patients, 34 (67%) had nodal clearance, while of the 26 N1 positive patients, 20 (77%) had nodal clearance after neo-adjuvant treatment. We then compared the radiological regression with the pathological response. A significant association was observed between RECIST criteria response with pCR and MPR (Table 4) .

**Table 4:** Association between radiologic and pathological response

RECIST	pCR	non-pCR	*p*-value	MPR	non-MPR	*p*-value
CR	3	0	**0.003**	3	0	**0.019**
PR	24	42		41	25	
SD	0	6		1	5	

CR, complete response; PR, partial response; SD, stable disease

### Pathological response and clinical parameters

Based on pathological response, the patients were divided into a pCR group and a non-pCR group, as well as a MPR group and a non-MPR group. We then explored the correlation between the pre-operative factors and pathological reactions (Table 5). No significant association was observed between the pathological response with sex, disease stage, differentiation degree, immunotherapy drug or neo-adjuvant dose. However, we found that patients with age more than 65 were more likely to have a pathological response of MPR (*P* = 0.011) or pCR (*P* = 0.001). In addition, MPR occurred more often among patients with single node positive (*P* = 0.036) and SCC (*P* = 0.039).

**Table 5:** Characteristics classified by pathological response

	pCR	non-pCR	*p*-value	MPR	non-MPR	*p*-value
Age			**0.001**			**0.011**
≥ 65 < 65	20 7	16 32		27 18	9 21	
Sex			1			0.931
Male Female	25 2	44 4		42 3	27 3	
Smoking status			0.767			0.819
Current or former smoker Never smoked	25 2	42 6		41 4	26 4	
Location			0.604			0.209
LU LL RU RM RL	10 2 8 2 5	13 7 17 1 10		18 4 12 2 9	5 5 13 1 6	
lober atelectasis			0.436			0.298
Yes No	2 25	8 40		4 41	6 24	
Stage at baseline			0.76			0.784
IIb IIIa IIIb	10 14 3	14 27 7		15 25 5	9 16 5	
Tumor stage			0.899			0.512
T1 T2 T3	11 12 4	17 23 8		19 20 6	9 15 6	
Nodal stage			0.407			0.488
N1 N2	11 16	15 33		17 28	9 21	
Nodal positive			0.265			**0.036**
Multiple Single	4 23	14 34		7 38	11 19	
Histology			0.081			**0.039**
Adenocarcinoma Squamous-cell carcinoma	2 25	13 35		5 40	10 20	
Differentiation degree			0.083			0.059
Poor Moderate or well	9 18	26 22		17 28	18 12	
Immunotherapy			0.707			0.919
tislelizumab pembrolizumab	18 9	34 14		31 14	21 9	
Neo-adjuvant doses			0.88			0.992
2 3 4	20 5 2	38 7 3		35 7 3	23 5 2	

### Survival

**Fig. 1:**
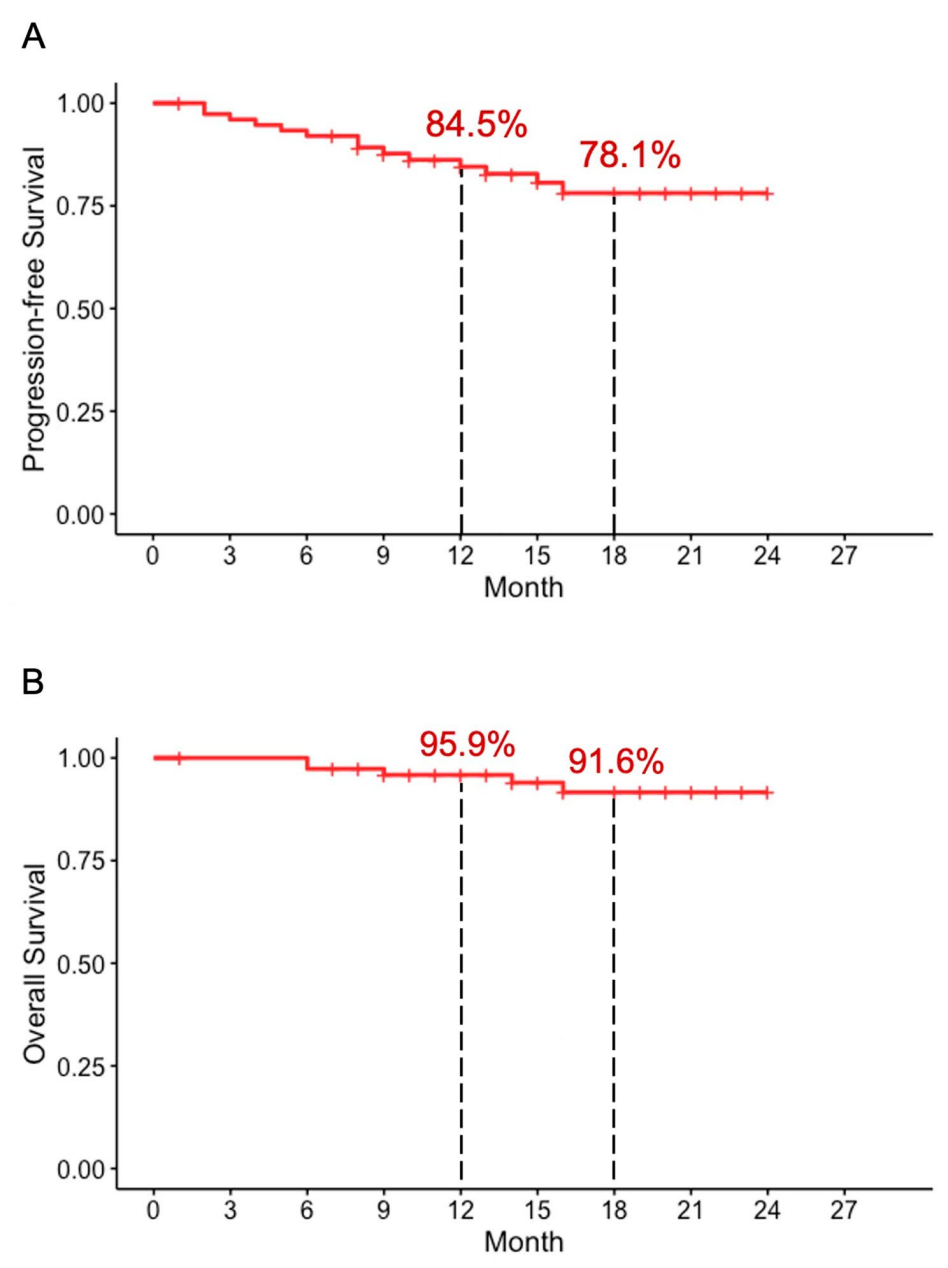
Kaplan-Meier curves of progression-free survival (**A**) and overall survival (**B**) in all enrolled patients

At the time of data cutoff, with a median post-operative follow-up of 16.1 (range, 7–24) months, 70 (93.3%) patients were alive and 61 (81.3%) patients had no evidence of recurrence. 3 patients died of brain metastasis, 1 patient died of trachea metastasis and 1 patient died of multiple metastasis. In the entire patient cohort, median duration of progression-free survival and overall survival was not reached. The PFS and OS were 84.5% and 95.5% at 12 months and 78.1% and 91.6% at 18 months (Fig. 1).

**Fig. 2:**
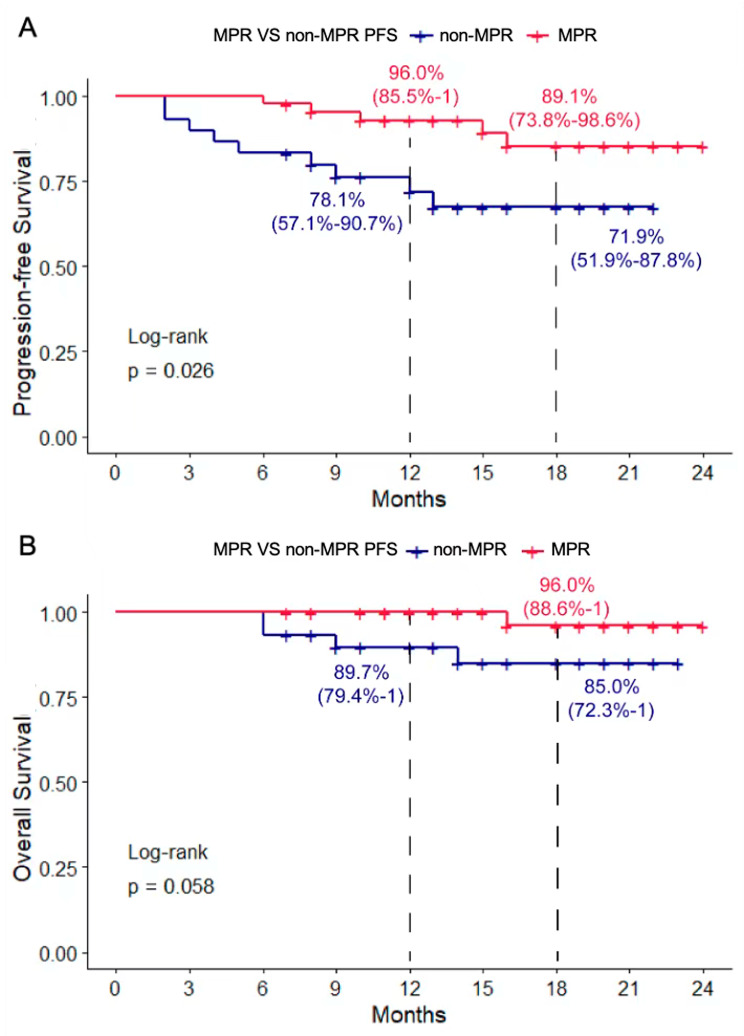
Progression-free survival (**A**) and overall survival (**B**) in patients with major pathological response (MPR) and without major pathological response (non-MPR)

We then analyzed the PFS and OS at the time points of 12 months and 18 months between different groups of pathological response and nodal status. Of 27 patients with pCR, the OS at 12 and 18 months was 100%, compared with 93.5% and 86.7% in the non-pCR patients. The PFS of these two groups was 96% and 89.1% versus 78% and 71.8%. No significant difference of PFS and OS was observed between the pCR and non-pCR groups (Sup Fig. 2). Among the MPR patients, the OS was 100% and 96%, compared with 89.7% and 85% in the non-MPR group. The PFS of MPR patients was 92.9% and 85.3%, which was significantly higher than that in non-MPR patients, 72% and 68.5% respectively (*P* = 0.026) (Fig. 2).

**Fig. 3:**
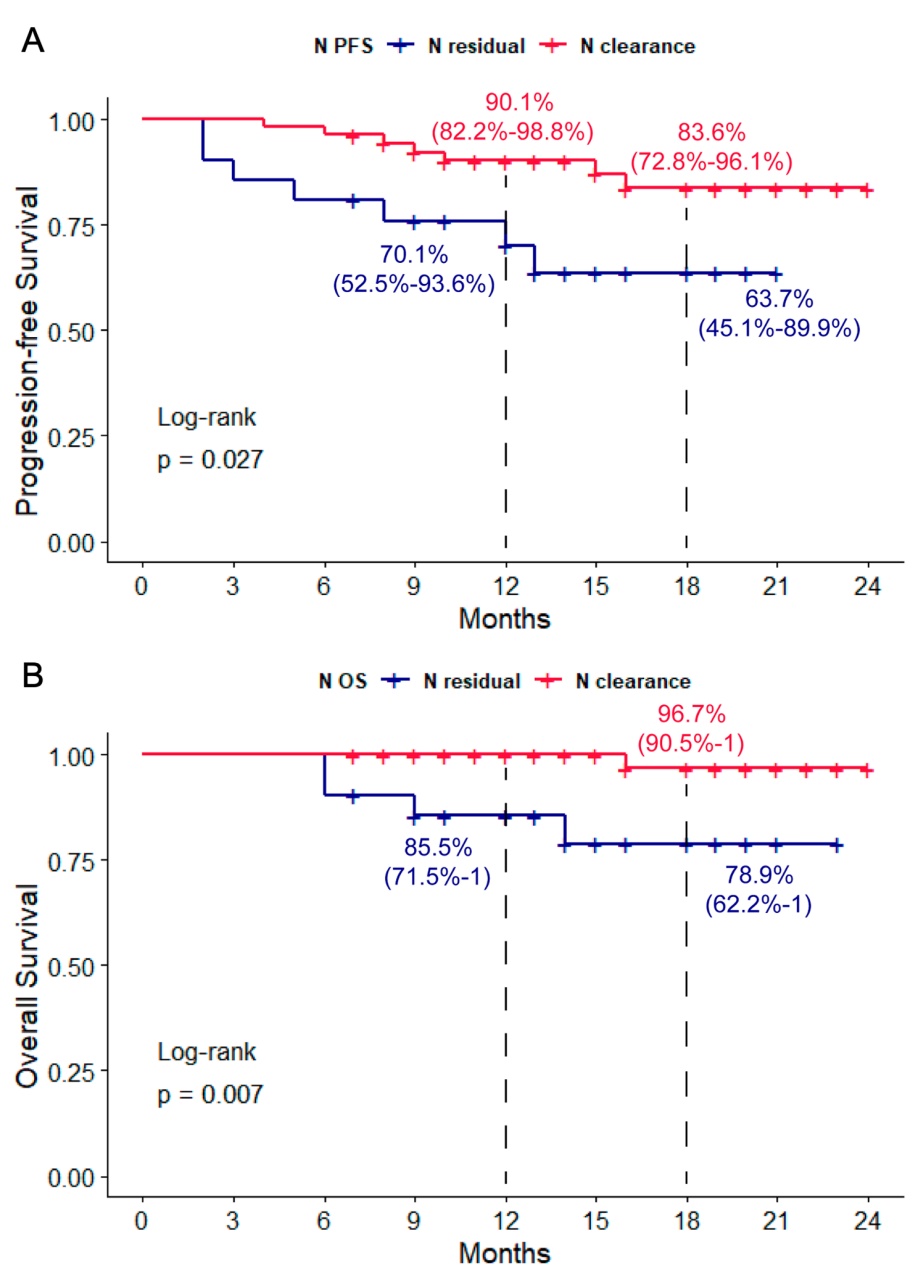
Progression-free survival (**A**) and overall survival (**B**) in patients with nodal clearance and nodal residual

With respect to the nodal status, 90.1% and 83.6% patients were progression free at 12 and 18 months in patients with nodal clearance, compared with 70% and 63.3% in nodal residual patients. The OS of nodal clearance patients was 100% and 96.6%, compared with 85.4% and 78.8% in nodal residual patients. Significant difference was identified in both PFS and OS between patients with different nodal status after neo-adjuvant treatments (*P* = 0.027 and *P* = 0.007) (Fig. 3).

Additionly, we analyzed the PFS and OS based on the original TNM stage and PD-L1 expression. Compared to IIIb, patients with IIb and IIIa were observed to have better survival events after the neo-adjuvant treatment plus surgery (Sup Fig. 3). The evaluation of PD-L1 expression was available in the biopsy samples of 52 patients. Though no statiscal difference was observed, the patients with PD-L1 positive exhibited a better tendency of survival (Sup Fig. 4).

Also, we evaluated the adjuvant therapy effect on the survival of PFS and OS. No significant difference was identified between two groups (Sup Fig. 5).

## Disscussion

In this retrospective study, we analyzed the combination of chemotherapy and immunotherapy in the locally advanced NSCLC, which showed a safe and feasible clinical outcome.

Immune checkpoint inhibitors (ICIs, PD-L1/PD-1 inhibitors) alone or in combination with chemotherapy have been approved for first-line use in metastatic or advanced NSCLC [16]. Several clinical trials concerning the efficacy of neo-adjuvant ICIs or chemo-immunotherapy are currently being evaluated. There are theoretical advantages by using the ICIs in neo-adjuvant setting. The primary tumor and draining lymph nodes are critical for antigen presentation. And pre-operative induction of immunotherapy provides an early opportunity to treat micrometastatic disease PD-1 blockade [7]. As proved in the metastatic NSCLC, the combination of immunotherapy and chemotherapy significantly improved both PFS and OS [17]. Likewise in the resectable early-stage NSCLC, the Checkmate-816 outcomes indicated longer survival and better pathological response by neo-adjuvant use of immunotherapy plus chemotherapy [10].

OS is the gold standard of cancer treatment effect. As the prolonged survival time, reliable surrogate indicators for efficacy and survival are needed in the neo-adjuvant context. Pathological assessments of the primary tumor and lymph nodes after neo-adjuvant therapy provide rapid analysis of treatment effect. Currently, MPR and pCR are frequently proposed as the surrogate end points for immunotherapy-based treatment efficacy, based on the data from neo-adjuvant chemotherapy trials in NSCLC [9]. But the accuracy of MPR and pCR to the predict long-time survival still remains to be validated by the ongoing neo-adjuvant trials. In our study, 27 (36%) patients who achieved complete pathological response were all alive at the time of data collection. The PFS in patients with MPR was significantly higher than the non-MPR patients, which was consistent with the previous published trials [18]. Our data supports the role of the pathological response as a potential survival surrogate.

In metastatic NSCLC trials, the PD-L1 expression and TMB are proved to be predictors of ICI efficiency [19]. However no similar correlation between these two biomarkers and treatment response was observed in several neo-adjuvant resectable NSCLC studies [20]. Till now, no pre-treatment predictor is supposed to be predictive for favorable responses to ICI treatment. In our study, we analyzed the relationship between clinical parameters and pathological response. Our data showed that the patients with elder ages were more likely to have better treatment response. Consistent with other study [21], we also found that a higher proportion of patients with squamous-cell carcinoma (66.7%) had MPR than those with adenocarcinoma (33.3%). In addition, we revealed that multiple lymph node metastasis at baseline was a negative factor for the pathological response. 38 (66.7%) patients with single nodal positive had MPR compared with 7 (38.9%) patients with multipe nodal positive (*P* = 0.036). The association between radiologic response and pathological response was reported conflicting. Our data showed 39.1% patients with ORR had pCR and 63.8% patients with ORR had MPR, compared with 0% and 16.7% patients with SD disease (*P* = 0.003 and *P* = 0.019).

As all the patients enrolled in the study were of N1/N2 lymph node positive, we then evaluated the nodal status after neo-adjuvant treatment. Previous researches have proved that the the downstaging or clearance of metastasis lymph node was associated with long-term survival after neoadjuvant chemotherapy or chemoradiation therapy [22, 23]. In our data of neo-adjuvant chemo-immunotherapy, 67% patients with N2 positive and 77% patients with N1 positive had nodal clearance. Compared to the nodal residual cohort, the patients with nodal clearance had better PFS and OS (*P* = 0.027 and *P* = 0.007).

By analyzing the adverse events, our data showed the neo-adjuvant chemo-immunotherapy was well tolerated, which was consistent with previous reports [21]. Although most patient experienced adverse events, grade 3 or 4 events only occurred in 8 patients, of which 1 patient surgery delayed and no peri-operative death happened. The intra-operative technical difficulty is another main concern after neoadjuvant treatment. Perihilar or lobar fbrosis are common phenomenon which are related to infammatory treatment responses at the primary tumor and involved nodal stations. In our study, 68% patients received thoracoscopic surgery and all the patients received R0 resection. No additional surgical complications attributed to the neo-adjuvant treatment were observed. Our data indicated that the patients could safely undergo operations after neo-adjuvant chemo-immunotherapy.

The present study had some limitations. By design, the patients included in this study was from a single center and the data was retrospective. Selection bias might existed. In addition, the sample size recruited in the study was small and the postoperative follow-up period was still short.

## Conlusion

Our findings suggested that the regimen of immunotherapy plus chemotherapy was safe and feasible in the locally advanced NSCLC with N1/N2 lymph node positive. The patients with elder age, SCC and single node positive had better pathological response. After neo-adjuvant chemo-immunotherapy, the nodal clearance was a positive prognostic factor.

### Electronic supplementary material

Below is the link to the electronic supplementary material.

Supplementary Material 1

Supplementary Material 2

## Data Availability

All data generated or analysed during this study are included in this published article and its supplementary information files.
