# Supplementary material for: Neo-adjuvant chemotherapy plus immunotherapy in resectable N1/N2 NSCLC
Source: BMC Cancer. 2023 Dec 21;23:1260. doi: 10.1186/s12885-023-11745-x (PMC10734172; doi:10.1186/s12885-023-11745-x)
Supplement: Supplementary file 1 — Supplementary Material 1 [file 12885_2023_11745_MOESM1_ESM.docx]

| **Table S1 Neo-adjuvant therapy** | |
| --- | --- |
| Immune checkpoint inhibitor  Tislelizumab  pembrolizumab | 52 (71%)  23 (29%) |
| Doses of neo-adjuvant therapy  2 doses  3 doses  4 doses | 58 (77%)  12 (16%)  5 (7%) |
| Duration from final treatment to surgery  Median (range) | 32.6 (24-71) |
| RECIST status  CR  PR  SD | 3 (4%)  66 (88%)  6 (8%) |

| **Table S2 Adjuvant therapy** | | | | |
| --- | --- | --- | --- | --- |
|  | Patients | Doses | | |
|  | 50 | 1 dose | 2 doses | >2 doses |
| ICIs+ Chemo | 35 | 13 | 22 | 0 |
| ICIs | 12 | 2 | 6 | 4 |
| Chemo | 3 | 1 | 2 | 0 |
