# Supplementary material for: Neo-adjuvant chemotherapy plus immunotherapy in resectable N1/N2 NSCLC
Source: BMC Cancer. 2023 Dec 21;23:1260. doi: 10.1186/s12885-023-11745-x (PMC10734172; doi:10.1186/s12885-023-11745-x)
Supplement: Supplementary file 2 — Supplementary Material 2 [file 12885_2023_11745_MOESM2_ESM.docx]

**Sup Figure 1**


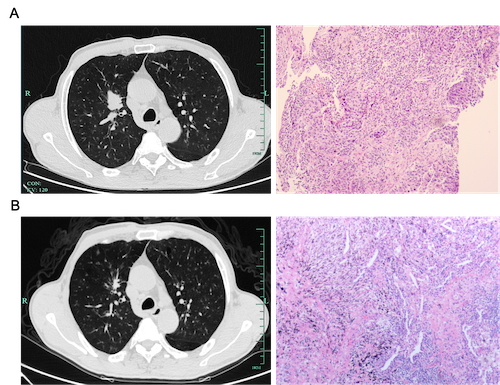


Sup Figure 1. Radiological and pathological response after neo-adjuvant chemo-immunotherapy. (A) Before treatment, (B) After treatment.

**Sup Figure 2**


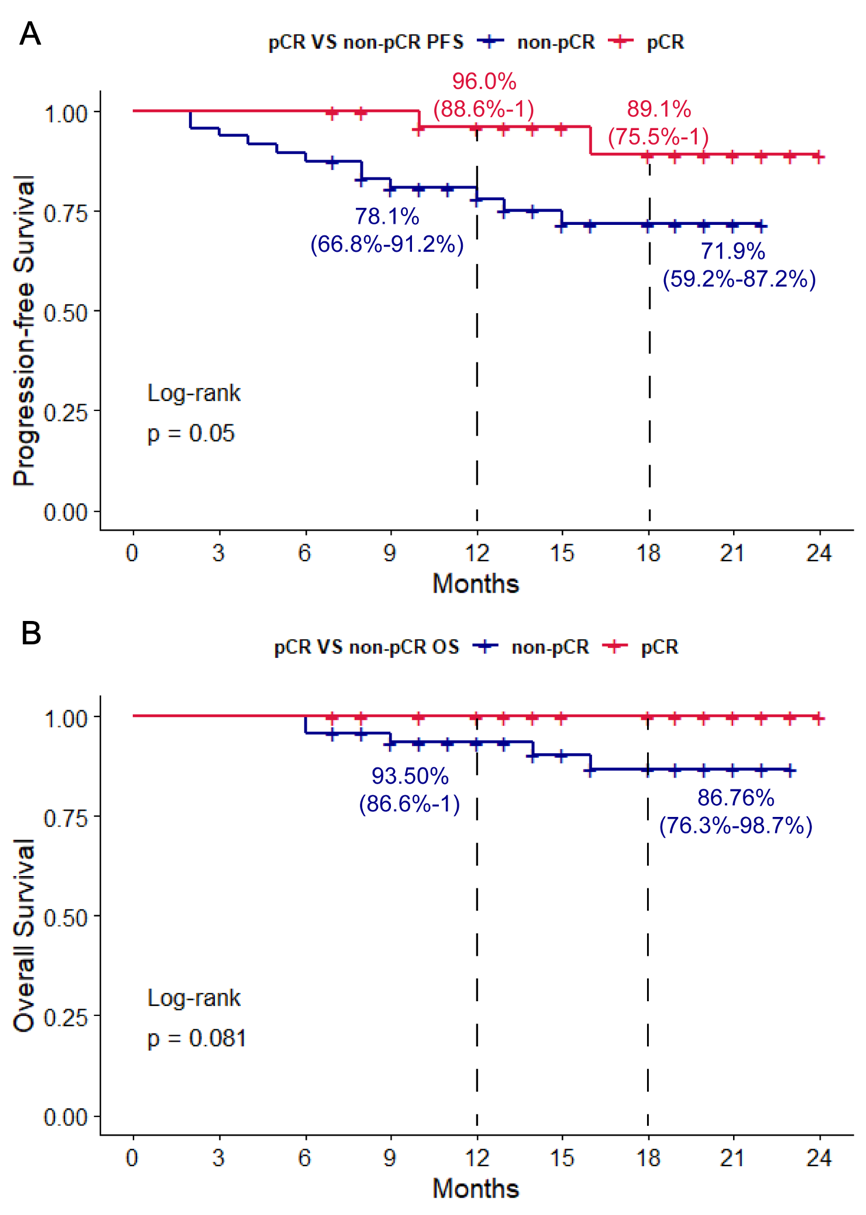


Sup Figure 2. Progression-free survival (A) and overall survival (B) in patients with complete pathological response (pCR) and incomplete pathological response (non-pCR).

**Sup Figure 3**


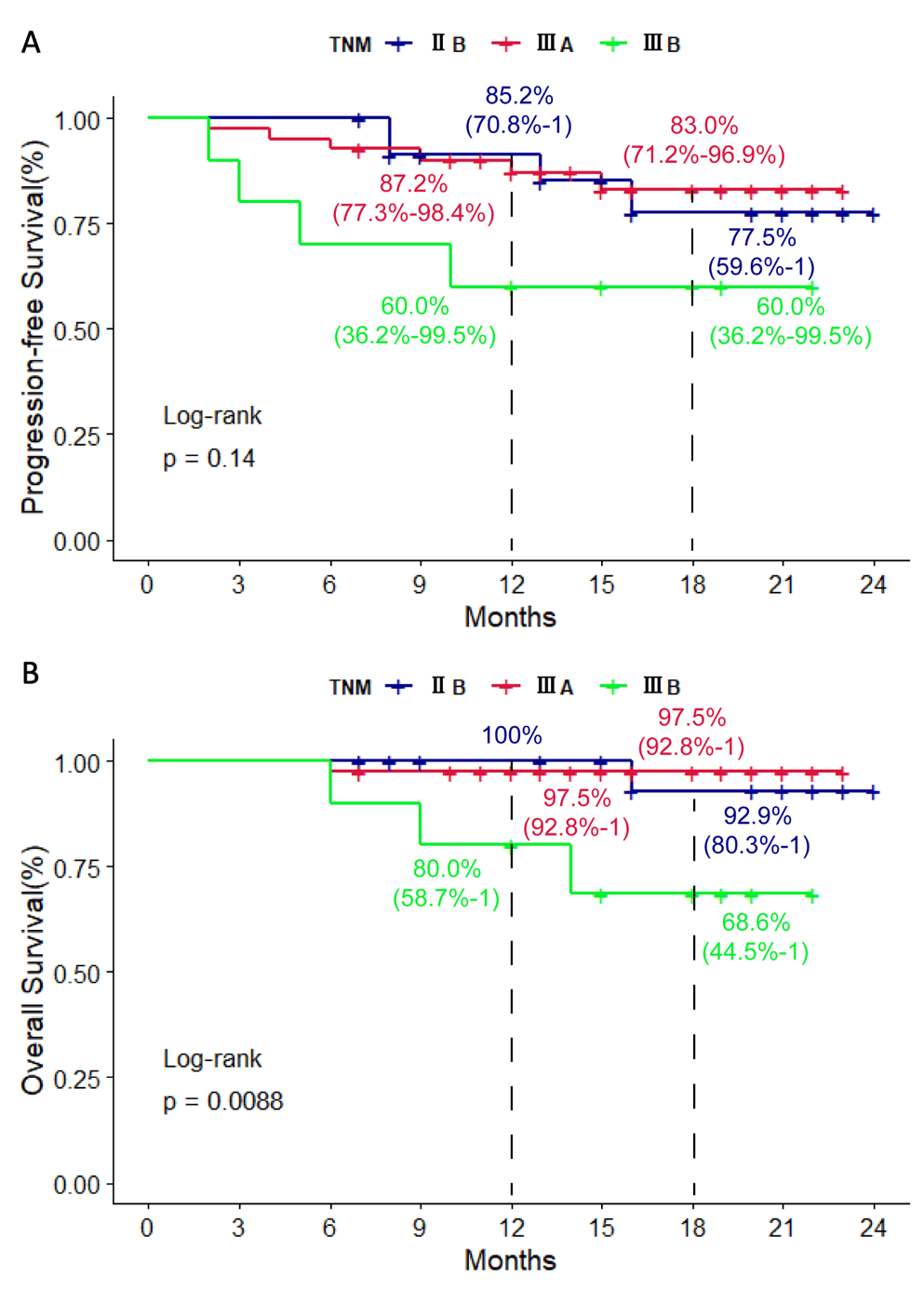


Sup Figure 3. Progression-free survival (A) and overall survival (B) in patients with different TNM stage (IIb, IIIa, IIIb) after neo-adjuvant treatment plus surgery.

**Sup Figure 4**


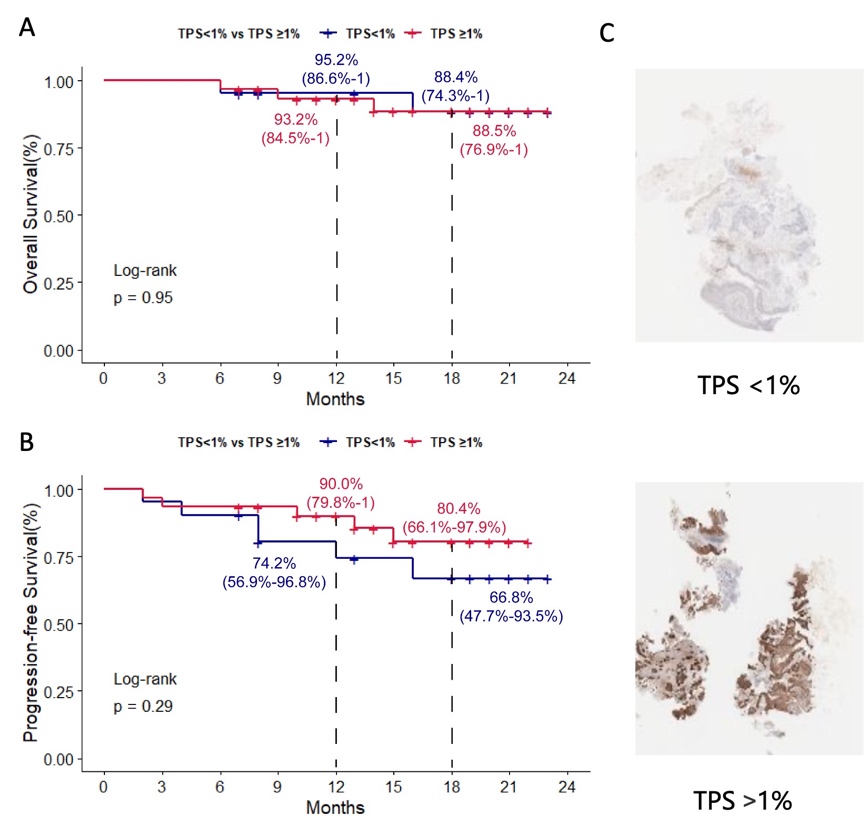


Sup Figure 4. Progression-free survival (A) and overall survival (B) in patients with PD-L1 TPS <1% and ≥1%. The representative IHC images were shown in (C).

**Sup Figure 5**


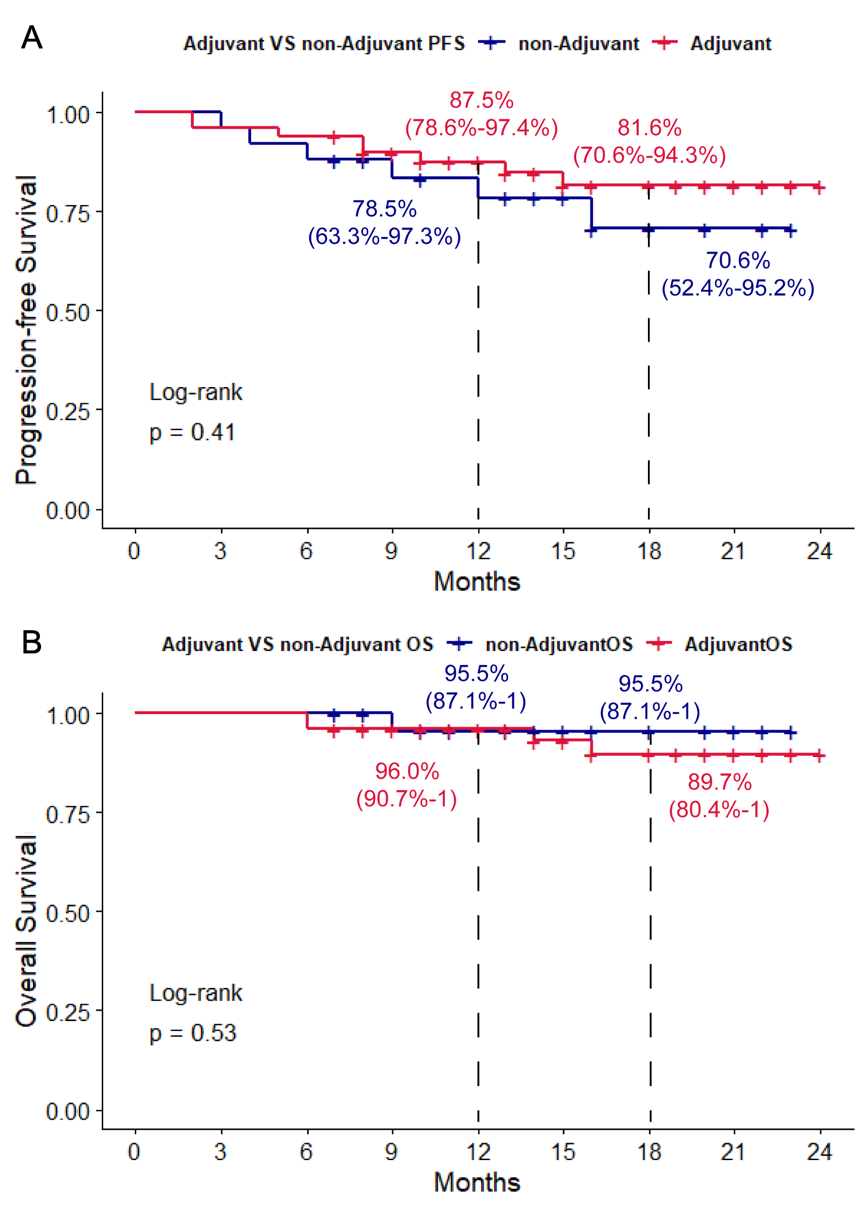


Sup Figure 5. Progression-free survival (A) and overall survival (B) in patients who received adjuvant treatment in comparison with patient without adjuvant treatment.
